# Prevalence and burden of diabetes mellitus-related symptoms in patients with type 2 diabetes mellitus: A cross-sectional study

**DOI:** 10.51866/oa.416

**Published:** 2023-12-05

**Authors:** Jonathan Yuet Han Tan, Chirk Jenn Ng

**Affiliations:** 1 MD, M.FamMed, Klinik Kesihatan Sungai Manila, D/A32 Pejabat Kesihatan Kawasan Sandakan,Tingkat 6, Rumah Persekutuan Sandakan, Sandakan, Sabah, Malaysia, Email: tanyuethan@gmail.com; 2 MBBS, MMed Family Medicine, PhD, Health Services & Systems Research, Duke NUS Medical School, 8 College Road, Singapore.

**Keywords:** T2DM, DSC-R, Symptom burden, Prevalence, Patient's satisfaction

## Abstract

**Introduction:**

Type 2 diabetes mellitus (T2DM) is a significant non-communicable disease in Malaysia, with a prevalence of 18.1%, per the National Health and Morbidity Survey. This study aimed to determine the prevalence and burden of diabetes mellitus-related symptoms and whether these symptoms were addressed by primary care doctors.

**Methods:**

This 1-month cross-sectional study was conducted at an urban hospital-based primary care clinic in Malaysia. Patients with T2DM were recruited using systematic random sampling. Participants answered a self-administered questionnaire adapted from the Diabetes Symptom Checklist-Revised, which evaluated the sociodemographic characteristics, burden of diabetes mellitus-related symptoms in the past month and post-consultation feedback about symptoms. Data were analysed using SPSS.

**Results:**

Four hundred eighteen participants were included, yielding a response rate of 97.7%. Hyperglycaemia was the most prevalent symptom, with 48.1% of the participants reporting a frequent need to empty their bladder. Most participants experienced a low symptom burden, so 56.7% did not report their symptoms to their doctors. The participants who reported their symptoms had a higher symptom burden. Among them, 97.5% indicated that their doctors addressed their symptoms. Approximately 78% reported satisfaction and good coping skills when their symptoms were addressed.

**Conclusion:**

Hyperglycaemia was the most prevalent diabetes mellitus-related symptom among the patients with T2DM. The symptom burden was generally low, so most patients did not report their symptoms to their doctors. Those who reported their symptoms had a higher symptom burden. Further studies must explore why patients do not report their symptoms and how doctors address patients’ symptoms.

## Introduction

Diabetes mellitus substantially impacts the quality of life (QoL) of patients by affecting their physical, mental and social well-being.^1^ Patients with type 2 diabetes mellitus (T2DM) may experience physical symptoms such as acute or chronic pain, fatigue or neuropathy and psychological symptoms such as depression, sleep disturbances or emotional disability.^2^

T2DM-related symptoms can be as burdensome as the disease itself. The prevalence of these symptoms ranges from 3% to 48%.^2^ Patients with T2DM have been reported to have a poorer QoL than their healthy counterparts.^3^

Despite their impact on QoL, diabetes mellitus-related symptoms are often not reported by patients. Most studies are focused on hypoglycaemia among patients with T2DM most likely because it is the most burdensome symptom.^3^ In contrast, limited studies have investigated the burden of other diabetes mellitus-related symptoms among patients with T2DM.

In Malaysia, the prevalence of T2DM is high.^1^ Accordingly, it is important to study the burden of diabetes mellitus-related symptoms and whether these symptoms are addressed by doctors, especially among patients with a high symptom burden, to consequently improve their QoL.

Patients with T2DM have a poorer health-related QoL (HRQoL) than their healthy counterparts. Sociodemographic characteristics, disease control and symptoms are determinants of the HRQoL of patients with T2DM.^4^ A substantial number of patients report a poorer HRQoL owing to pain/discomfort, mobility problems, anxiety/depression, reduced activity performance and impaired ability for self-care.^5^ Some patients with T2DM also develop complications such as ischaemic heart disease, stroke and neuropathy, further worsening their HRQoL.^6^ Older patients with T2DM tend to have a higher symptom burden than their younger counterparts. One study showed that patient-reported symptoms in older patients were risk factors for hospitalisation and emergency department visits.^7^

A study conducted by the American Diabetes Association found that 56% of patients with T2DM experienced at least one diabetes mellitus-related symptom in the past 12 months.^8^ Patients with T2DM may also have emotional and psychological needs that must be addressed.^9^ Accordingly, the symptom burden is a patient concern.^10^ Addressing diabetes mellitus-related symptoms may improve patients’ emotional and psychological wellbeing.

It is also essential to know whether doctors address patients’ symptoms because such symptoms are as important as their concerns. Two-thirds of patients have been shown to worry that their symptoms might represent a serious illness. Accordingly, identifying and addressing patients’ concerns are a crucial part of the patient-centred approach.^11^

Current clinical practice focuses on the control of HbAlc levels and prevention of complications of T2DM rather than control of symptoms. Patients with T2DM may have symptoms, which may burden them. This study sought to conduct a proper clinical assessment and provide symptom relief to patients. Symptoms can be an indicator of disease progress or a complication of the disease, such as atherosclerosis.^12^ Diabetes mellitus-specific symptoms are important predictors that facilitate a patient-centred approach. This study then aimed to identify the prevalence and burden of diabetes mellitus-related symptoms among patients with T2DM and the degree of symptom management by primary care doctors.

## Methods

### Design

A prospective cross-sectional study was conducted from 1 October 2019 to 30 November 2019 at the Department of Primary Care Medicine in University Malaya Medical Centre, a tertiary hospital located in Kuala Lumpur, Malaysia.

### Participants

Patients with T2DM who were aged >18 years and able to understand either English or Malay language were included in the study. Those who were cognitively impaired were excluded from the study.

### Instrument

A self-administered questionnaire adapted from the Diabetes Symptom Checklist-Revised (DSC-R), with an additional section assessing participant demographics, was used. Its English version was translated to Malay language by two independent translators who were proficient in both languages. The Malay version was reviewed by an expert panel. This version underwent backward translation to English by two other independent translators who were also proficient in both languages. The questionnaire had two sections: preconsultation and post-consultation. The preconsultation section assessed the participants’ sociodemographic and clinical characteristics and diabetes mellitus-related symptoms. The modified DSC-R consisted of 34 diabetes mellitus-related symptoms, requiring the participants to respond either ‘yes’ or ‘no’ if they had any of those symptoms in the past 4 weeks. Those who responded ‘yes’ for each symptom were required to rate their symptom burden on a Likert scale ranging from 1 (‘not at all troublesome’) to 5 (‘extremely troublesome’). For the post-consultation section, the participants indicated their feedback regarding their consultation.

### Pilot study

A pilot study was conducted among 30 participants prior to the actual data collection to identify any issues with the questionnaire and the recruitment process. These participants were able to understand the questionnaire and, on average, took about 20 min to complete it. No changes were made to the questionnaire after the pilot study.

Subsequently, patients with T2DM were recruited via systematic random sampling. Eligible participants received the preconsultation questionnaire before their consultation and the post-consultation questionnaire after their consultation with their doctors.

### Main study

A total of 1602 patients with T2DM were identified at the triage counter of the hospital within 1 month. These patients were randomly and systematically recruited, with one selected for every three. A total of 534 patients were selected. Among them, 54 were unable to read or understand English or Malay; 11 refused to participate; and nine had a cognitive impairment. Consequently, 460 patients remained and agreed to participate; they were given the Patient information sheet to read, and the consent form to sign. Once the consent form was signed, the participants were asked to complete the questionnaire. A total of 418 participants completed and returned the questionnaires. The primary outcome of this study was the prevalence of symptoms during the past 4 weeks. The secondary outcome was the symptom burden, which was assessed using a Likert scale. Other outcomes included symptoms reported to doctors and whether doctors addressed such symptoms.

**Figure 1:**
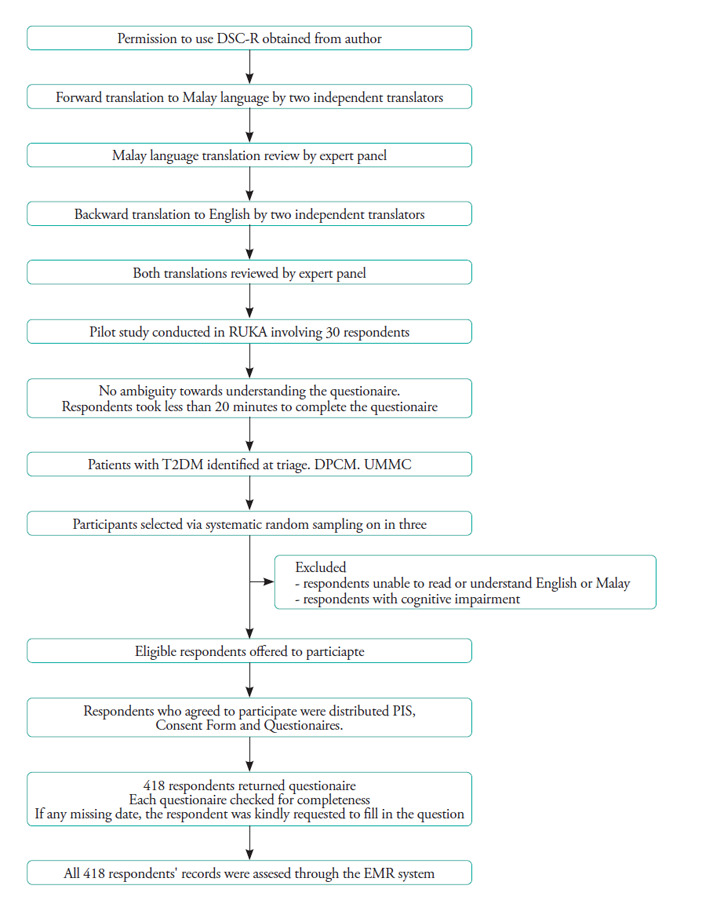
Flowchart of the study and data collection process.

### Data analysis

Data were analysed using SPSS version 23.0 by IBM, Chicago, United States of America. Descriptive statistics were used to describe the sociodemographic and clinical data of the participants. Categorical variables were presented as percentages and frequencies and continuous variables as means with standard deviations (SDs). The independent variables were the sociodemographic and clinical characteristics, while the dependent variables were the symptom score, subscale score and post-consultation feedback. The association of the sociodemographic and clinical characteristics with the prevalence of symptoms and the symptom score was also evaluated.

### Ethical considerations

Ethical approval was obtained from the University Malaya Medical Ethics Committee before commencement of the study (MREC ref. no.: 201973-7602). Written informed consent was obtained from all participants.

## Results

A total of 471 patients were eligible for inclusion, of whom 11 refused to participate, and 42 who consented did not return the questionnaires. This yielded a response rate of 88.7%. The participants had a mean age of 63 years. Approximately 55.5% were women, and 41.8% were Malays. Most participants (80.6%) had T2DM for more than 5 years, with a mean HbA1c level of 7.98% (Table 1).

**Table 1:** Sociodemographic and clinical variables of the respondents (N=418).

Sociodemographic and clinical variables	n (%)
**Sex (n=4l8)**	
Male	186 (44.5)
Female	232 (55.5)
**Age (year) (n=4l8)**	
30 and below	2 (0.5)
31-40	16 (3.8)
41-50	34 (8.1)
51-60	86 (20.6)
61-70	179 (42.8)
71-80	89 (21.3)
81 and above	12 (2.9)
**Ethnicity (n=4l8)**	
Malay	174 (41.6)
Chinese	100 (24.0)
Indian	138 (33.0)
Others	6 (1.4)
**Highest educational qualification (n=4l8)**	
Primary school and below	60 (14.4)
Secondary school	280 (67.0)
Pre-university	49(11.7)
Tertiary	29 (6.9)
**Duration of T2DM (year) (n=4l8)**	
0-5	81 (19.4)
6-10	148 (35.4)
11-15	95 (22.7)
16-20	71 (17.0)
21-25	10 (2.4)
26-30	8 (1.9)
31 and above	5 (1.2)
**Other disease (n=4l8)**	
Hypertension	317 (75.8)
High cholesterol	283 (67.7)
Heart disease	44 (10.5)
Stroke	13 (3.1)
Kidney disease	18 (4.3)
**Medications for DM (n=4l8)**	
None	3 (0.7)
Insulin	97 (23.2)
Oral medications	318 (76.1)
**HbAlc level (%) (n=378)**	
6.5 and below	92 (24.3)
6.6-8.0	134 (35.5)
8.1 and above	152 (40.2)
**eGFR (n=389)**	
Normal (90mL/min/1.73m^2^. and above)	196 (50.4)
Stage 2 CKD	130 (33.4)
Stage 3 CKD	44(11.3)
Stage 4 CKD	8(2.1)
Stage 5 CKD	11 (2.8)

*eGFR = Estimated gromerular filtration rate

### Prevalence and burden of diabetes mellitus-related symptoms

The prevalence of diabetes mellitus-related symptoms ranged from 4.1% to 48.1%. The most commonly reported symptoms were frequent need to empty the bladder (48.1%), numbness of the hands (43.5%), lack of energy (42.6%) and numbness of the feet (40.9%).

Most symptoms were reported to be slightly to moderately troublesome (mean score=2.00–2.58). The most troublesome symptoms were related to hyperglycaemia: polyuria (mean score=2.58), thirst (mean score=2.45) and lack of energy (mean score=2.44) (Table 2). Similarly, among the subscale symptoms, those related to hyperglycaemia scored the highest (0.91), followed by symptoms related to psychology–fatigue (0.64) and neurology–sensory (0.52) (Table 3).

**Table 2:** Prevalence and burden of diabetes mellitus-related symptoms.

Occurring symptom	n (%)	Mean (SD)
Frequent need to empty the bladder	201 (48.1)	2.58 (0.95)
Numbness of the hands	182 (43.5)	2.30 (0.86)
Lack of energy	178 (42.6)	2.44 (0.80)
Numbness of the feet	171 (40.9)	2.42 (0.85)
Very thirsty	150 (35.9)	2.45 (0.86)
Dry mouth	146 (34.9)	2.12 (0.79)
Overall sense of fatigue	133 (31.8)	2.22 (0.86)
Drinking a lot	133 (31.8)	2.51 (0.96)
Sleepiness or drowsiness	119 (28.5)	2.12 (0.79)
Aching calves when walking	112 (26.8)	2.24 (0.84)
Increasing fatigue during the course of the day	103 (24.6)	2.00 (0.79)
Persistently blurred vision	98 (23.4)	2.00 (0.91)
Tingling sensations in the limbs at night	91 (21.8)	2.24 (0.69)
Difficulty concentrating	82 (19.6)	2.00 (0.77)
Shooting pains in the leg	66 (15.8)	2.23 (0.84)
Easily irritated or annoyed	65 (15.6)	2.22 (1.08)
Fatigue in the morning when getting up	59 (14.1)	2.19 (1.01)
Tingling or prickling sensations in the hands or fingers	54 (12.9)	2.04 (0.73)
Deteriorating vision	52 (12.4)	2.21 (0.67)
Difficulty paying attention	51 (12.2)	2.06 (0.61)
Shortness of breath during physical exertion	49 (11.7)	2.35 (1.01)
Tingling or prickling sensations in the lower legs	49 (11.7)	2.27 (0.73)
Moodiness	48 (11.5)	2.19 (0.87)
Burning pain in the calves at night	47 (11.2)	2.11 (0.81)
Fuzzy feeling in the head	46 (11.0)	1.76 (0.92)
Alternating clear and blurred vision	37 (8.9)	2.11 (0.61)
Palpitation or pounding in the heart region	36 (8.6)	1.78 (0.68)
Flashes or black spots in the field of vision	35 (8.4)	1.89 (0.80)
Pain in the chest or heart region	34 (8.1)	1.76 (0.82)
Odd feeling in the leg or feet when touched	30 (7.2)	2.03 (0.76)
Shortness of breath at night	27 (6.5)	1.85 (0.72)
Irritability just before a meal	25 (6.0)	1.92 (1.15)
Burning pains in the legs during the day	23 (5.5)	1.83 (0.94)
Sudden deterioration of vision	17 (4.1)	2.24 (1.09)

**Table 3:** Subscale scores.

Subscale (N=418)	Mean (SD)
Hyperglycaemia	0.91 (0.96)
Psychology-fatigue	0.64 (0.77)
Neurology-sensory	0.52 (0.57)
Psychology-cognitive	0.36 (0.60)
Neurology-pain	0.32 (0.57)
Hypoglycaemia	0.24 (0.59)
Ophthalmology	0.24 (0.51)

**Table 4:** Association of the mean subscale score with age, HbAlc level and DM duration.

Subscale	Psychology – fatigue	Psychology – cognitive	Neurology – pain	Neurology – sensory	Cardiology	Ophthalmology	Hypoglycaemia	Hyperglycaemia
Age (n=4l8)								
Pearson	0.62	0.009	0.006	0.008	-0.088	-0.065	-0.020	0.028
correlation	0.203	0.857	0.898	0.100	0.074	0.187	0.681	0.568
P-value								
HbAlc level (n=378)	**0.102**	0.058	0.075	0.066	**0.103**	0.083	0.082	0.054
Pearson correlation	**0.047**	0.257	0.145	0.203	**0.045**	0.106	0.113	0.294
P-value								
DM duration (n=4l8)	**0.133**			**0.168**		**0.134**		
Pearson correlation	**0.006**	0.064	-0.011	**0.000**	-0.029	**0.006**	0.035	0.093
		0.192	0.825		0.558		0.479	0.057
P-value								

### Management of symptoms by doctors

Approximately 83.5% of the participants had previously consulted their attending doctors. Among them, 38% discussed their symptoms with their doctors. Approximately 88.1% (n=l40) reported one to three symptoms, with a mean number of symptoms of 2.26 (SD=1.6) (Table 5). Nearly all participants (97.5%) indicated that their symptoms were addressed by their doctors; most were satisfied (89.3%) with how their symptoms were addressed and were confident (78.0%) in coping with their symptoms.

**Table 5:** Post-consultation review.

Post-consultation feedback	n (%)
Is this the first time you are seeing this doctor? (n=418) Yes No	69 (16.5) 349 (83.5)
Did you discuss your symptom with doctor? (n=418)	
Yes	159 (38.0)
No	237 (56.7)
Do not have symptom	22 (5.3)
How many symptoms did you discuss with the doctor? (n=159)	
1-3	140 (88.1)
4-6	15 (9.4)
7 and above	4 (2.5)
Did the doctor address your symptom(s)? (n=159)	
Yes	155 (97.5)
No	1 (0.6)
Not sure	3 (1.9)
Were you satisfied with how the doctor addressed your symptom(s)? (n=159)	
Not satisfied at all	2 (1.3)
Not satisfied	2 (1.3)
Neutral	13 (8.2)
Somewhat satisfied	68 (42.8)
Very satisfied	74 (46.4)
After consultation with the doctor, how confident are you in coping with your symptom(s)? (n=159) Not confident at all Not confident Neutral Confident Very confident	1 (0.6) 2 (1.3) 32 (20.1) 83 (52.2) 41 (25.8)

## Discussion

This study showed that the overall prevalence of diabetes mellitus-related symptoms and the prevalence of each symptom among the patients with T2DM were quite low and below 50%, respectively. The participants in this study were generally old with a long disease duration, but the prevalence of symptoms was lower than expected. The most commonly reported symptoms were related to hyperglycaemia, possibly reflecting disease control. Most symptoms were acknowledged and addressed satisfactorily by the doctors of the participants.

The prevalence of symptoms ranged from 4.1% to 48%, while the symptom burden score ranged from 1.76 to 2.58, reflecting a low symptom burden. These findings differ from the report by García et al., wherein the prevalence ranged from 14.1% to 67.6%.^13^ The authors found that a stronger perception of disease severity was associated with a higher symptom burden.^13^

In the present study, the low prevalence of symptoms among the participants could be attributed to the relatively average HbA1c level of 7.9%. Müller et al. showed that most patients with T2DM with hyperglycaemic symptoms had an HbA1c level above 8.9%.^14^ Similar to the present findings, the hyperglycaemic symptoms that were most prevalent were frequent urination and tiredness.^14^ Further, higher HbA1c levels were associated with a higher symptom burden.

Other complications of T2DM may also affect patients’ symptoms. Patients with cardiovascular or ophthalmological complications may have a higher symptom burden. Symptoms of hypoglycaemia may be more prevalent in patients on insulin; however, in this study, the score for this subscale was not significant. The patients with a longer T2DM duration tended to have a higher symptom. (Table 5)

Among the participants who discussed their symptoms with their doctors, 88.1% reported a total of one to three symptoms. Most of them (97.5%) indicated that their doctors addressed their symptoms. Notably, this study was conducted at a primary care clinic in a tertiary hospital in Malaysia, where patients’ expectations tend to be higher. Providing adequate time during consultations contributes to patient satisfaction.^15^

### Strengths and limitations

The strength of this study is that the findings are applicable to clinical practice. Patients with an advanced age and a higher HbA1c level may have a higher symptom burden.

There are several limitations noted in this study. This study was conducted in a single setting, limiting the generalisation of the findings to other settings in Malaysia. Further, the questionnaire was translated only to Malay. Many patients who were unable to read or understand English or Malay were then excluded from this study. Some patients with visual impairment or stroke would require assistance in completing the questionnaire. Another limitation is that the questionnaire was tested for its face and content validities only; it was not validated with other questionnaires such as the Diabetes Distress Scale and SF-36. Moreover, the symptoms were evaluated retrospectively for the past 4 weeks, so there was a possibility of recall bias. The post-consultation section mainly evaluated the symptoms based on consultation with the doctors. However, the symptoms may not be the primary concern of some patients. The data were also susceptible to recall bias.

Another limitation of this study is that the symptoms reported by the patients with T2DM may be attributed to diseases other than T2DM such as benign prostate hyperplasia (BPH) or overactive bladder syndrome (OAB). Liu et al. reported a high prevalence of OAB among patients with T2DM.^16^ In other studies, T2DM was found to be associated with BPH in men and bladder dysfunction in women. Berger et al. concluded that diabetic vascular damage may cause hypoxia, which may be involved in the pathogenesis of BPH.^17,18^ The association of T2DM with prostate or bladder disorder could explain the high prevalence of urinary symptoms among patients with T2DM.

## Conclusion

The prevalence and burden of diabetes mellitus-related symptoms among patients with T2DM are low. Optimisation of glycaemic control is important in reducing the symptom burden. A lower symptom burden results in fewer discussions of symptoms with doctors. Generally, patients with T2DM are satisfied with the management of their symptoms by their doctors.

## Data sharing statement

The data that support the findings of this study are available on request from the corresponding author. These data are not publicly available since they contain information that could compromise the privacy of the participants.

**How does this paper make a difference in general practice?**

- The findings can benefit individuals practising in the field of family medicine, especially in areas where non-communicable diseases are prevalent.
- The study can assist future researchers in conducting literature reviews.
- The study can help future researchers in generating ideas for research in the field of non-communicable diseases.
- The findings emphasise the importance of reporting symptoms to doctors.
- The findings can be used as a guide by healthcare practitioners in addressing patients’ symptoms.
